# Simultaneous Conduction and Valence Band Regulation of Indium-Based Quantum Dots for Efficient H_2_ Photogeneration

**DOI:** 10.3390/nano11051115

**Published:** 2021-04-26

**Authors:** Xiu-Ping Li, Rong-Jin Huang, Cong Chen, Tianduo Li, Yu-Ji Gao

**Affiliations:** Shandong Provincial Key Laboratory of Molecular Engineering, School of Chemistry and Chemical Engineering, Qilu University of Technology (Shandong Academy of Science), Jinan 250353, China; lixp2021@163.com (X.-P.L.); huangrongjin2021@163.com (R.-J.H.); chencong199889@163.com (C.C.); ylpt6296@vip.163.com (T.L.)

**Keywords:** artificial photosynthesis, bandgap engineering, quantum dots, transition metal doping

## Abstract

Indium-based chalcogenide semiconductors have been served as the promising candidates for solar H_2_ evolution reaction, however, the related studies are still in its infancy and the enhancement of efficiency remains a grand challenge. Here, we report that the photocatalytic H_2_ evolution activity of quantized indium chalcogenide semiconductors could be dramatically aroused by the co-decoration of transition metal Zn and Cu. Different from the traditional metal ion doping strategies which only focus on narrowing bandgap for robust visible light harvesting, the conduction and valence band are coordinately regulated to realize the bandgap narrowing and the raising of thermodynamic driving force for proton reduction, simultaneously. Therefore, the as-prepared noble metal-free Cu_0.4_-ZnIn_2_S_4_ quantum dots (QDs) exhibits extraordinary activity for photocatalytic H_2_ evolution. Under optimal conditions, the Cu_0.4_-ZnIn_2_S_4_ QDs could produce H_2_ with the rate of 144.4 μmol h^−1^ mg^−1^, 480-fold and 6-fold higher than that of pristine In_2_S_3_ QDs and Cu-doped In_2_S_3_ QDs counterparts respectively, which is even comparable with the state-of-the-art cadmium chalcogenides QDs.

## 1. Introduction

Artificial photosynthesis is regarded as a promising approach to convert solar energy into usable energy forms, such as molecular hydrogen (H_2_), to resolve the energy crisis and environment pollution [1,2,3,4,5]. Hence, synthesis of efficient and low-cost photocatalysts is with vital importance for solar-to-fuel conversion. The semiconductor quantum dots (QDs) which the size is less than their exciton Bohr radius in three dimensions have been attracted tremendous attention in this field owing their unique properties, such as large surface area, abundant surface active sites, multiple exciton generation, short charge transfer distance and so on [6,7,8,9,10,11]. Among the popular chalcogenide semiconductor-based QDs in artificial photocatalysis, the indium-based chalcogenides seem to be more promising photocatalysts due to their less toxic, noble metal-free as well as the potential visible light harvesting [12,13,14,15]. However, the photocatalytic H_2_ evolution activity of indium-based photocatalysts is relatively faint so far, especially compared with their cadmium chalcogenide analogs [16,17,18,19], so more works remained to be done in optimizing the properties of indium-based QDs. In general, though the bandgap of bulk In_2_S_3_ (~2.3 eV) is suitable for absorbing visible light, the bandgap of quantized indium chalcogenide nanoparticles is usually larger than 3.0 eV as a result of quantum confinement effect [19,20,21,22,23]. On the other hand, the more negative conduction band edge corresponds to the stronger thermodynamic driving force for proton reduction [24,25], which would result in the higher photocatalytic performance. To balance the above two contradictory effects of narrowing the bandgap and upshifting the conduction band edge, the conduction and valence band should be well designed.

In this contribution, we present that the conduction and valence band edge of indium chalcogenide-based QDs are regulated coordinately by introducing transition metal ions, Zn and Cu. The introduction of Zn ions could level up the conduction band by hybridizing Zn 4s4p and In 5s5p orbitals, while the doping of Cu would form a discrete level from Cu 3d above the valence band of pristine nanocrystals. As the upshift of valence band is obviously larger than that of conduction band, the bandgap of Cu-doped ZnIn_2_S_4_ QDs narrows remarkably to be suitable for absorbing visible light. And the upshift of conduction band by the introduction of Zn resulted in the higher thermodynamic driving force for proton reduction, prominently enhancing the photocatalytic H_2_ evolution activity of as-prepared QDs photocatalysts. With the assistance of Ni^2+^ and visible light irradiation, the Cu-doped ZnIn_2_S_4_ QDs could produce H_2_ efficiently with the rate of 144.4 μmol h^−1^ mg^−1^, 480-fold and 6-fold higher than that of pristine In_2_S_3_ QDs and Cu-doped In_2_S_3_ QDs, respectively. Under optimal conditions, more than 1000 μmol of H_2_ could be produced from 6.0 mL aqueous solution within 16 h irradiation, giving rise to the turnover number (TON) of more than 20260 per QD. The value is even comparable to the cadmium chalcogenide QDs-based photocatalytic systems (Table S1) [6,26]. Further, the apparent quantum yield (AQY) of Cu_0.4_-ZnIn_2_S_4_ QDs could reach 11.8% at 460 nm.

## 2. Materials and Methods

### 2.1. Materials

Indium acetate (99.99%), zinc acetate dihydrate (98%), copper(II) acetate monohydrate (99%), L-cysteine (98%), thioacetamide (99%), nickel acetate tetrahydrate (99.9%) and ascorbic acid (H_2_A, 99%) were purchased from Alfa Aesar Chemicals Co. Ltd. (Shanghai, China). Other chemicals were of analytical grade without further purification unless otherwise noted. The ultrapure water with 18.2 MΩ cm @ 25 °C was used throughout all the experiments.

### 2.2. Instruments and Characterizations

UV-vis spectra were measured with a Shimadzu UV-2600PC spectrophotometer (Shimadzu Corp., Kyoto, Japan). Fluorescence measurements were carried out with a Hitachi (model F-4600) spectrophotometer (Hitachi High-Tech Corp., Tokyo, Japan) at room temperature. TEM images were obtained on a JEM 2100 (JEOL Co. Ltd., Tokyo, Japan) operating at 200 kV. X-ray diffraction (XRD) pattern was obtained by using Bruker D8 Focus (Bruker Corp., Billerica, MA, USA) under Cu-K_α_ radiation. X-ray photoelectron spectroscopy (XPS) measurements were taken on an ESCALAB 250 spectrophotometer (ThermoFisher Scientific Corp., Waltham, MA, USA) with Al-K_α_ radiation. The binding energy scale was calibrated using the C 1s peak at 284.60 eV. All pH measurements were made with a Model pHS-3C meter (Mettler Toledo FE20, Mettler Toledo (Shanghai) Co. Ltd., Shanghai, China). The generated amount of H_2_ was characterized by GC analysis (GC-2014 Shimadzu, Shimadzu Corp., Kyoto, Japan) using N_2_ as the carrier gas with a molecular sieve column (5 Å; 30 m × 0.53 mm) and a thermal conductivity detector.

### 2.3. Synthesis of the Cu-Doped ZnIn_2_S_4_ and In_2_S_3_ QDs

The indium-based chalcogenides QDs were synthesized through a simple hydrothermal method referring to the previous reported method with some revisions [22]. Taking the Cu_0.4_-ZnIn_2_S_4_ QDs (0.4 represents the precursor molar ratio of Cu with Zn) for example, 0.85 mmol of zinc acetate, 1.70 mmol of indium acetate, 0.34 mmol of copper acetate monohydrate and 3.25 mmol of L-cysteine were dissolved in 30 mL of ultrapure water and adjusted the pH to 9.0. Following this, 3.25 mmol of thioacetamide was added and the mixture was heated to 110 °C for 4 h after vigorous stirring. After reaction, the solution was swiftly cooled to room temperature, and then the product was precipitated and purified with isopropanol and water. The obtained Cu-doped ZnIn_2_S_4_ QDs were dispersed in 40 mL of ultrapure water for further use.

The QDs with different contents of Cu and/or Zn were synthesized by regulating the amount of copper and/or zinc precursors (copper/zinc acetate), while the amount of thioacetamide was also changed with stoichiometric ratio. For the pristine In_2_S_3_ QDs, the reaction was carried out with the same expect for the absence of copper and zinc precursors.

### 2.4. General Procedure for Photocatalytic H_2_ Evolution

The photocatalytic reactions were performed under 460 nm LEDs irradiation with the Ni^2+^ as cocatalysts and H_2_A as the electron donor. Generally, 1.6 mg of QDs, 10 μg of nickel acetate tetrahydrate and 400 mg of ascorbic acid were added to a 20 mL of Pyrex tube. The total reaction volume of the mixture was 6.0 mL by adding ultrapure water and the pH of the solution was adjusted to 5.2 by HCl or NaOH. Prior to irradiation, the sample was sealed and de-aerated by bubbling N_2_ for 10 min to remove the dissolved oxygen, and then 1.0 mL of CH_4_ was injected the system to serve as internal standard. The amount of evolved H_2_ gas was quantified by comparing the area ratio of CH_4_ to H_2_ and the response factor of CH_4_/H_2_ in the gas chromatography (GC).

### 2.5. The Calculated of AQY for Cu_0.4_-ZnIn_2_S_4_ QDs

For the optimal photocatalytic H_2_ evolution, the irradiation area was 3.0 cm^2^ by 460 nm LEDs (light intensity 100 mW·cm^−2^). The calculation of apparent quantum efficiency (*Φ*) is according to the following equation:

$$\Phi =\frac{2\times n_{H_{2}}}{N}\times 100\%$$

wherein *n_H_2__* is the amount of photo-generated H_2_ and *N* is the corresponding amount of incident photon. The amount of incident photon was calculated from the irradiation time, the irradiation area and the illumination power. It showed that about 5.5 mL of H_2_ was generated after 1.0 h irradiation. From the combined measurements of the amount of photo-generated H_2_ and the corresponding amount of incident photon, the apparent quantum yield was calculated to be 11.8%.

## 3. Results and Discussion

### 3.1. Microstructure Characteristics and Composition Analysis

**Figure 1:**
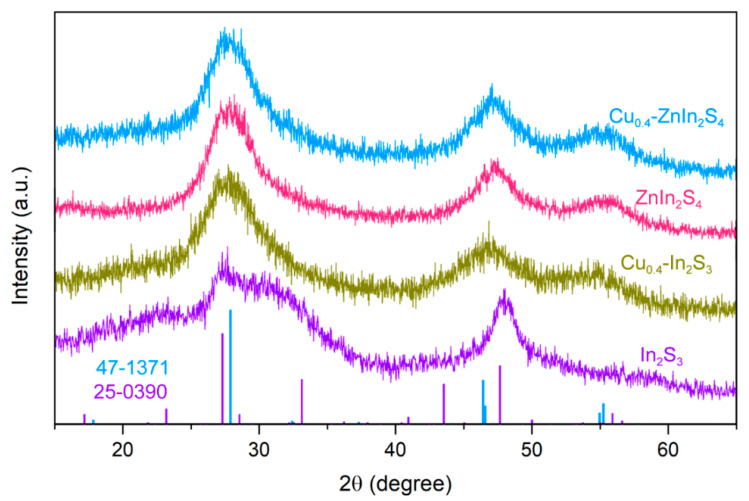
The XRD patterns of In_2_S_3_, Cu_0.4_-In_2_S_3_, ZnIn_2_S_4_ and Cu_0.4_-ZnIn_2_S_4_ QDs.

The photocatalysts with different contents of Zn and/or Cu decorated In_2_S_3_ QDs were synthesized by regulating the species and amount of precursors. According to the photocatalytic H_2_ evolution performance, the as-prepared pristine In_2_S_3_, ZnIn_2_S_4_, Cu_0.4_-In_2_S_3_ and Cu_0.4_-ZnIn_2_S_4_ QDs were selected for the structure and morphology characterizations. As shown in Figure 1, the diffraction peaks of the pristine In_2_S_3_ can be indexed as the tetragonal phase In_2_S_3_ (JCPDS no. 25-0390) [27]. However, though the XRD patterns change clearly after the co-decoration of Zn and Cu ions (Cu_0.4_-ZnIn_2_S_4_), the synthesized counterparts exhibit three characteristic peaks at about 28.0°, 47.5° and 55.3°, which can be assigned to (112), (024) and (132) lattice plane of tetragonal phase of Cu_0.412_In_0.412_Zn_0.175_S (JCPDS no. 47-1371) [20]. What’s more, the diffraction peaks of Zn or Cu decorated In_2_S_3_ QDs (ZnIn_2_S_4_ or Cu_0.4_-In_2_S_3_) are well corresponding to that of Cu_0.4_-ZnIn_2_S_4_, demonstrating the identical crystal structure of the above QDs. Additionally, the varied contents of Zn or Cu hardly shifted the diffraction peaks of the as-prepared indium-based chalcogenide QDs (Figure S1), which probably owes to the similar radius of Zn^2+^ (0.74 Å), Cu^+^ (0.74 Å) and In^3+^ (0.76 Å) [28,29], demonstrating the preservation of crystal structure during the transition metal ions decoration.

**Figure 2:**
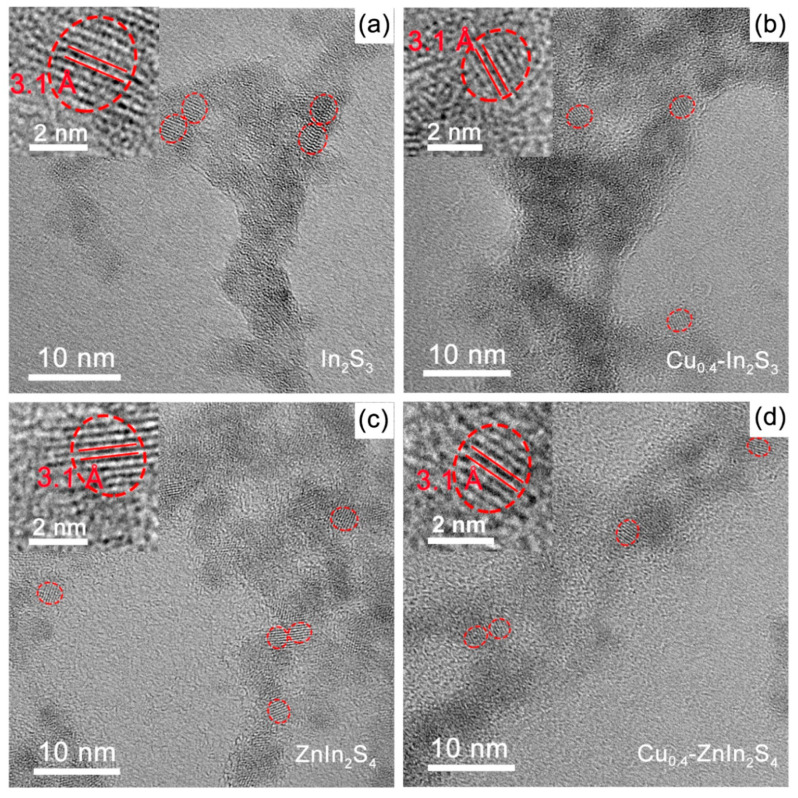
The TEM images of (**a**) In_2_S_3_, (**b**) Cu_0.4_-In_2_S_3_, (**c**) ZnIn_2_S_4_ and (**d**) Cu_0.4_-ZnIn_2_S_4_ QDs. The insets show the corresponding high-resolution TEM images.

The size and morphology of the synthesized various QDs were also investigated. According to Debye–Scherrer formula, the broad diffraction peaks implied the ultra-small size of the nanocrystals in three dimensions [30,31]. While the similar full width at half maximum (FWHM) of different contents of Zn and/or Cu decorated In_2_S_3_ QDs indicated the approximate size (Figure 1). It should be mentioned that FWHM of pristine In_2_S_3_ QDs was smaller than the other QDs, which implied the slightly larger size of In_2_S_3_ QDs. Indeed, the diameters in terms of XRD patterns for In_2_S_3_, Cu_0.4_-In_2_S_3_, ZnIn_2_S_4_ and Cu_0.4_-ZnIn_2_S_4_ QDs along the (024) plane is 3.8 nm, 2.6 nm, 2.6 nm and 3.1 nm, respectively (the difference of size could be ignored in the photocatalytic activity comparison owing to the seriously faint photocatalytic H_2_ evolution of In_2_S_3_ QDs). The diameters of various QDs were further characterized by the transmission electron microscope (TEM) images. As shown in Figure 2a, the In_2_S_3_ QDs displayed the near-spherical nanocrystals with the diameter of about 3.9 nm, while the other three QDs (Figure 2b–d) were also near-spherical nanocrystals with the diameter ranging from 2.7~3.0 nm, slightly smaller than the pristine In_2_S_3_ QDs. What’s more, the high-resolution TEM (HR-TEM) images revealed that the lattice spacing of all the QDs was 3.1 Å [20,32,33], which was corresponding to the (112) plane of tetragonal phase of In_2_S_3_, ZnIn_2_S_4_ or their doped counterparts, further demonstrating that the crystal structure of tetragonal phase was well preserved after the introduction of Zn and/or Cu ions into the In_2_S_3_ QDs.

**Figure 3:**
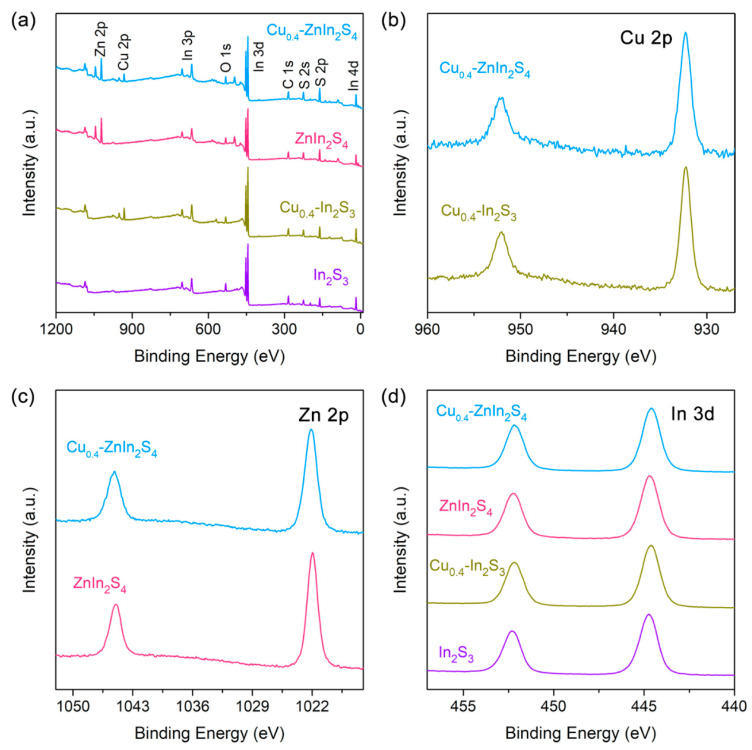
(**a**) The XPS survey spectra of In_2_S_3_, Cu_0.4_-In_2_S_3_, ZnIn_2_S_4_ and Cu_0.4_-ZnIn_2_S_4_ QDs. The high-resolution XPS spectra of (**b**) Cu 2p, (**c**) Zn 2p and (**d**) In 3d in various QDs, respectively.

To gain more insight into the chemical composition and valence state of metal elements in the prepared various QDs, the X-ray photoelectron spectroscopy (XPS) was explored. The peaks of Cu, Zn, In and S elements were clearly observed in the XPS survey spectrum of Cu_0.4_-ZnIn_2_S_4_ QDs [19,34,35], while the In_2_S_3_, ZnIn_2_S_4_ and Cu_0.4_-In_2_S_3_ also clearly manifested their respective peaks without the presence of other metal elements (Figure 3a), suggesting the rational chemical composition in the designed QDs. Furthermore, the binding energy of Cu 2p_3/2_ and Cu 2p_1/2_ in both Cu_0.4_-In_2_S_3_ and Cu_0.4_-ZnIn_2_S_4_ QDs was at 932.3 eV and 952.1 eV (Figure 3b), suggesting the monovalent Cu in the QDs, which could be further demonstrated by the absence of satellite peak [36]. The decrease of valence state of Cu probably owes to the reduction by L-cysteine. What’s more, the binding energies of Zn 2p (1022.1 eV for 2p_3/2_ and 1045.2 eV for 2p_1/2_) and In 3d (444.6 eV for 3d_5/2_ and 452.2 eV for 3d_3/2_) in Cu_0.4_-ZnIn_2_S_4_ QDs (Figure 3c,d), suggested that the chemical states of Zn and In are +2 and +3, respectively, which were in good agreement with the previous reports on Zn-In-S ternary semiconductors [37,38]. Additionally, the features and positions of In 3d, Zn 2p and S 2p peaks (Figure 3c,d and Figure S2) remained the same in the four QDs, indicating the similar lactic framework and coordination environment of the indium-based chalcogenide QDs. Combining all the above results, we believe that by regulating the species and amount of precursors, the Zn and/or Cu decorated In_2_S_3_ QDs with the identical crystal structure and similar size have been successfully prepared.

### 3.2. Photocatalytic H_2_ Evolution

**Figure 4:**
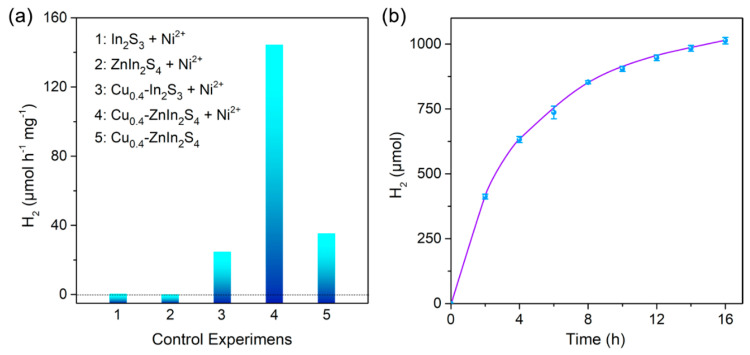
(**a**) Comparison of H_2_ evolution of Cu_0.4_-ZnIn_2_S_4_ QDs with In_2_S_3_, ZnIn_2_S_4_ and Cu_0.4_-In_2_S_3_ QDs under the in situ loaded Ni^2+^ as cocatalysts, and photocatalytic activity comparison of Cu_0.4_-ZnIn_2_S_4_ QDs with/without cocatalysts. (**b**) Long-time photocatalytic H_2_ evolution of Cu_0.4_-ZnIn_2_S_4_ QDs under optimal conditions. The photocatalytic reaction was performed with 1.6 mg of QDs, 10 μg of nickel acetate tetrahydrate and 400 mg of ascorbic acid dispersed in 6.0 mL aqueous solution at pH 5.2, using 460 nm LEDs irradiation. Error bars represent the mean ± s.d. of multiple independent experiments.

The photocatalytic activity of the as-prepared QDs were investigated by using the QDs as absorber and in situ loaded Ni^2+^ as cocatalysts. It indicated that without the introduction of Cu, there was no H_2_ to be detected for the ZnIn_2_S_4_ QDs, and only trace amount of H_2_ was produced for In_2_S_3_ QDs under 460 nm LEDs irradiation (Figure 4a). However, the photocatalytic H_2_ evolution activity dramatically increased after the doping of Cu for both In_2_S_3_ and ZnIn_2_S_4_ QDs, probably owing to the outstanding visible light harvesting by the narrowed bandgap. Therefore, the photocatalytic H_2_ evolution activities for different contents of Cu decorated QDs were further explored. As shown in Figure S3, the photocatalytic H_2_ evolution activity of both Cu-doped In_2_S_3_ and ZnIn_2_S_4_ QDs increased with the increased Cu contents at first and then decreased. This is due that the introduction of Cu would decrease the bandgap of QDs, which resulted in the enhanced photocatalytic activity. While the excess introduction of Cu would lead to the decreased redox driving force as well as the formation of Cu defects for non-radiative carrier recombination centers, which was unfavorable for the photocatalytic H_2_ evolution.

The photocatalytic H_2_ evolution rate for optimal Cu-doped ZnIn_2_S_4_ (Cu_0.4_-ZnIn_2_S_4_) QDs could reach to 144.4 μmol h^−1^ mg^−1^, which was nearly 480-fold and 6-fold higher than that of pristine In_2_S_3_ and Cu_0.4_-In_2_S_3_ QDs under identical conditions respectively (Figure 4a), implying the significant impact of Zn introduction for the enhancement of photocatalytic performance. Apparently, the photocatalytic H_2_ evolution rate is even comparable to the state-of-the-art cadmium chalcogenides QDs [26,39]. Control experiments also demonstrated that without the Ni^2+^, the photocatalytic activity would decrease by three fourths, indicated the vital role of cocatalysts. Long-time H_2_ evolution performance of the Cu_0.4_-ZnIn_2_S_4_ QDs was also examined under optimal conditions. As shown in Figure 4b, the rate was decreased with the irradiation time probably owing to the photocurrosion, however, ~1013 μmol of H_2_ could be obtained from 6.0 mL aqueous solution within 16 h irradiation, giving the TON of 20260 per QD. And the AQY of Cu_0.4_-ZnIn_2_S_4_ QDs could reach 11.8% at 460 nm.

### 3.3. Mechanism for Photocatalytic H_2_ Production

**Figure 5:**
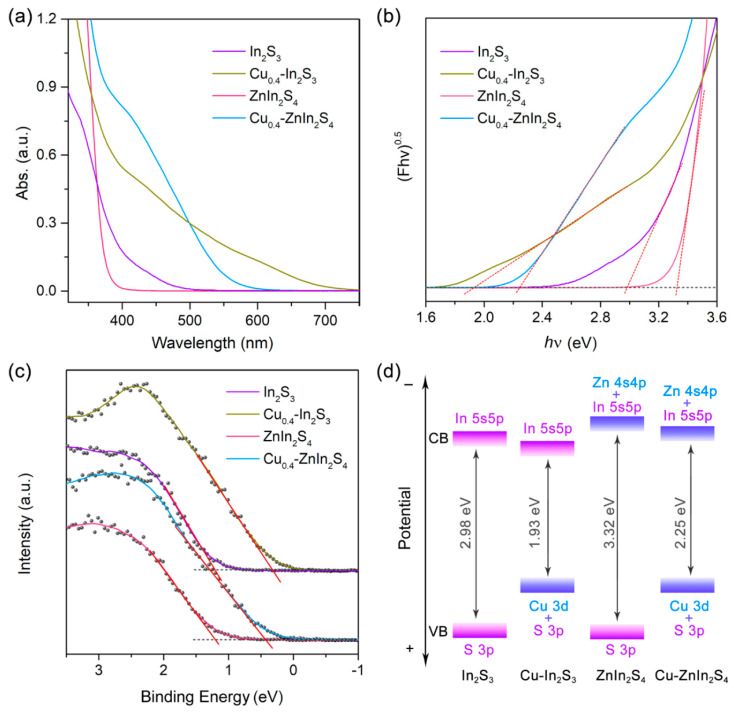
(**a**) The UV- vis absorption spectra, (**b**) corresponding Tauc plots, (**c**) XPS valence band spectra and (**d**) the corresponding band position alignment of In_2_S_3_, Cu_0.4_-In_2_S_3_, ZnIn_2_S_4_ and Cu_0.4_-ZnIn_2_S_4_ QDs.

To shed light on the effect of the introduction of transition metal ions, the UV-visible absorption spectra of various QDs were carried out. As shown in Figure 5a, the In_2_S_3_ QDs exhibited faint absorption for visible light, which was corresponding to their negligible photocatalytic activity. As the intrinsic bandgap of bulk In_2_S_3_ is 2.3 eV [40,41,42], the faint visible light absorption of the as-prepared In_2_S_3_ QDs was mainly owing to broadening bandgap resulted by the quantum confinement effect. Further investigation indicated that the introduction of Zn and Cu elements played the opposite effect for the In_2_S_3_ QDs [43]. The absorption onset of ZnIn_2_S_4_ QDs was less than 400 nm, implied the broader bandgap after the introduction of Zn. However, the Cu-doped In_2_S_3_ QDs displayed the robust visible light harvesting and the absorption tail could rise up to 700 nm, implied the narrower bandgap. What’s more, the doping of Cu would also narrow the bandgap of ZnIn_2_S_4_ QDs, and the absorption onset of Cu_0.4_-ZnIn_2_S_4_ QDs redshifted to 600 nm [44]. Therefore, the introduction of Cu makes the main contribution for the bandgap narrowing.

Although the optical density of Cu_0.4_-ZnIn_2_S_4_ was slightly higher than that of Cu_0.4_-In_2_S_3_ at 460 nm (Figure 5a), the difference of absorption ability hardly resulted in the 6-fold enhancement of photocatalytic activity. Therefore, the band positions of the four QDs were further investigated by Tauc plot and XPS valence band spectra. Figure 5b revealed that the bandgap of In_2_S_3_, ZnIn_2_S_4_ and their Cu-doped counterparts were 2.98 eV, 3.32 eV, 1.93 eV and 2.25 eV, respectively, which was consistent with the results of absorption spectra. While the valence band edges of In_2_S_3_ and ZnIn_2_S_4_ were close, at 1.20 V and 1.22 V (vs. NHE) respectively (Figure 5c), which was owing that the valence band of the chalcogenide semiconductors were mainly composed by S 3p orbitals [45]. However, the introduction of Cu ions would create a Cu dopant level above the pristine valence band edge [3,46], causing their obvious upshift (*ca.* 0.8 V). Combined with the difference in bandgap, the conduction band edges of In_2_S_3_ and ZnIn_2_S_4_ could be calculated at −1.78 V and −2.10 V, indicated the 0.32 V upshift after the introduction of Zn, which could also be observed for the comparison of Cu_0.4_-In_2_S_3_ and Cu_0.4_-ZnIn_2_S_4_ (Figure 5d). This is because that the conduction band minimum is mainly composed of hybrid d and sp orbitals of the metal cations [47], therefore, the introduction of Zn would result in the change of conduction band from In 5s5p to more negative hybrid orbitals of In 5s5p and Zn 4s4p [46,48]. The upshift of the conduction band edges gave rise to a higher thermodynamic driving force of proton reduction, and hence remarkably accelerating the H_2_ evolution rate in water splitting.

**Figure 6:**
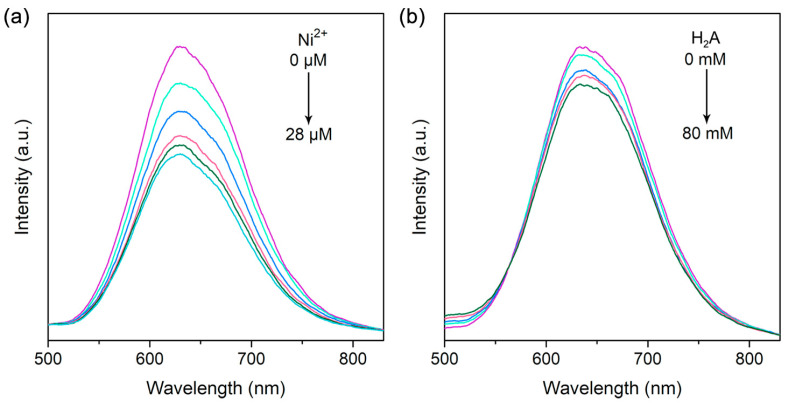
Steady-state emission quenching of Cu_0.4_-ZnIn_2_S_4_ QDs with gradual addition of (**a**) Ni^2+^ and (**b**) H_2_A.

Next, we employed the steady-state emission quenching experiments to evaluate the charge transfer process. Take the Cu_0.4_-ZnIn_2_S_4_ QDs as example, excitation of QDs at 430 nm would result in strong luminescence at 630 nm (Figure 6a), which roughly corresponded to the bandgap energy of Cu_0.4_-ZnIn_2_S_4_ QDs, indicating that this is the band edge emission of Cu_0.4_-ZnIn_2_S_4_ QDs. However, the emission intensity was dramatically quenched with the adding of Ni^2+^ (Figure 6a), indicating that the binding of Ni^2+^ with the QDs would result in the electron transfer from the QDs to Ni^2+^ and consequently inhibit the radiative recombination of photogenerated electron-hole pairs [49]. What’s more, after adding electron donor, H_2_A, into the QDs aqueous solution, the emission would also be quenched obviously (Figure 6b), demonstrating the hole transfer from QDs to H_2_A [50].

**Figure 7:**
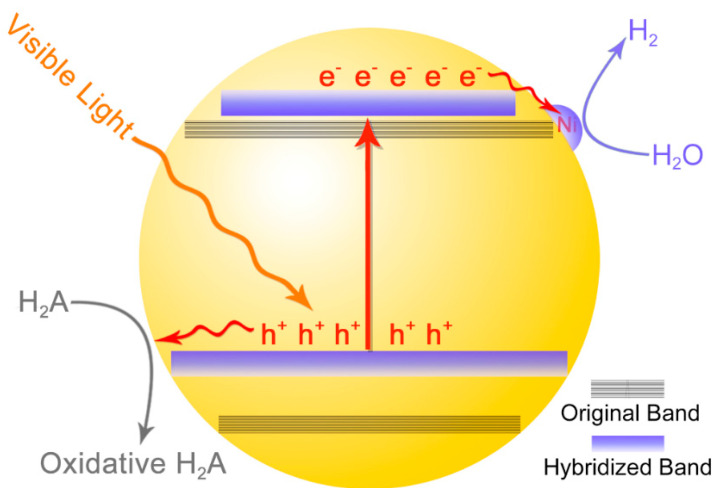
The transformation of conduction/valence band of In_2_S_3_ QDs after the decoration of Zn and Cu, as well as the photocatalytic H_2_ evolution mechanism of the Cu_0.4_-ZnIn_2_S_4_ QDs.

On the basis of the above results, we proposed that the introduction of Zn and Cu ions would regulate the conduction and valence band of indium chalcogenide QDs, respectively. As the upshift of valence band edge was significantly larger than that of conduction band edge, the bandgap of Cu doped QDs was clearly narrowed and gave rise to the robust visible light harvesting. Therefore, the photocatalytic activity of QDs remarkably enhanced after the doping of Cu. On the other hand, with the upshift of the conduction band edge, the thermodynamic driving force of proton reduction raised, thus the Cu-doped ZnIn_2_S_4_ QDs exhibited the superior photocatalytic activity. Under visible light irradiation, the photogenerated electron of Cu_0.4_-ZnIn_2_S_4_ QDs would transfer to the surface Ni species, the H_2_ evolution cocatalysts, which would assist the proton reduction and formation of H_2_. Simultaneously, the hole transferred to the surface of QDs and oxidized the electron donor to accomplish the whole reaction (Figure 7).

## 4. Conclusions

In summary, we have regulated the bandgap and conduction/valence band levels of indium-based chalcogenides QDs simultaneously by introducing transition metal ions to construct efficient and noble metal-free photocatalysts. The bandgap is mainly determined by the doping of Cu, which could remarkably upshift the valence band edge. While the introduction of Zn would slightly enhance the conduction band level and provide higher driving force for proton reduction. Therefore, the as-prepared Cu-doped ZnIn_2_S_4_ QDs exhibit outstandingly higher photocatalytic performance. Under the optimal conditions, the Cu_0.4_-ZnIn_2_S_4_ QDs could produce H_2_ with the rate of 144.4 μmol h^−1^ mg^−1^, which is even comparable to the state-of-the-art cadmium chalcogenide QDs. We believe that this approach has given a much deeper recognition on the band engineering, which can be extended to related systems as an effective strategy for the design of photocatalysts.

## Data Availability

The data presented in this study are available on request from the corresponding author.

## Supplementary Materials

The following are available online at https://www.mdpi.com/article/10.3390/nano11051115/s1, Table S1: Comparison of photocatalytic H_2_ evolution with reported cadmium chalcogenide QDs-based systems; Figure S1: The XRD patterns of Cu-doped Zn-In-S QDs (the ratio in the graph represents the molar ratio of Cu:Zn:In); Figure S2: The high-resolution XPS spectra of S 2p of In_2_S_3_, Cu_0.4_-In_2_S_3_, ZnIn_2_S_4_ and Cu_0.4_-ZnIn_2_S_4_ QDs; Figure S3: The photocatalytic H_2_ evolution comparison of different contents of Cu doped (a) In_2_S_3_ and (b) ZnIn_2_S_4_ QDs.
