# Supplementary material for: Simultaneous Conduction and Valence Band Regulation of Indium-Based Quantum Dots for Efficient H2 Photogeneration
Source: Nanomaterials (Basel). 2021 Apr 26;11(5):1115. doi: 10.3390/nano11051115 (PMC8146827; doi:10.3390/nano11051115)
Supplement: Supplementary file 1 [file nanomaterials-11-01115-s001.zip › nanomaterials-1196544-supplementary.pdf]

Supplemental Information

Simultaneous Conduction and Valence Band Regulation of Indium-Based Quantum Dots for Efficient H_2 Photogeneration

Xiu-Ping Li †, Rong-Jin Huang †, Cong Chen, Tianduo Li and Yu-Ji Gao *

Shandong Provincial Key Laboratory of Molecular Engineering, School of Chemistry and Chemical Engineering, Qilu University of Technology (Shandong Academy of Science), Jinan 250353, China; lixp2021@163.com (X.-P.L.); huangrongjin2021@163.com (R.-J.H.); chencong199889@163.com (C.C.); ylpt6296@vip.163.com (T.L.)

* Correspondence: yjgao@qlu.edu.cn

† These authors contributed equally to this work.

1. Table S1

Table S1. Comparison of photocatalytic H_2 evolution with reported cadmium chalcogenide QDs-based systems.

Photocatalysts	Cocatalysts	Light Source	Donor	TON vs. QD	Time (h)	Refer.
**CdTe QDs**	[FeFe]-H_2ase	>420 nm	H_2A	157.6	10	[1]
**CdSe QDs**	[FeFe]-H_2ase	>400 nm	H_2A	3 206	80	[2]
**CdSe QDs**	Ni-DHLA	520 nm	H_2A	1 200 000	360	[3]
**CdTe QDs**	Co^{2+}	>400 nm	H_2A	219 100	70	[4]
**CdSe QDs**	Ni^{2+}	>400 nm	IPA	15 340	20	[5]
**CdS QDs**	Co^{2+}	>420 nm	Na_2SO_3	29 000	88	[6]
**CdSe/ZnS QDs**	Cobaloxime	>400 nm	TEOA	>10 000	10	[7]
**CdS QDs**	Ni^{2+}	>400 nm	glycerol	38 405	20	[8]
**ZnS modified CdSe QDs**	None	450 nm	H_2A	440 000	40	[9]
**CdSe QDs**	ZnSe	>400 nm	H_2A	~5000	12	[10]
**$CdSe/Zn_{1-x}Fe_xS$ QDs**	FeS	405 nm	TEA	600 000	172 h	[11]
**$Cu_{0.4}$-$ZnIn_2S_4$ QDs**	Ni^{2+}	460 nm	H_2A	20 260	16 h	This work

2. Figure S1

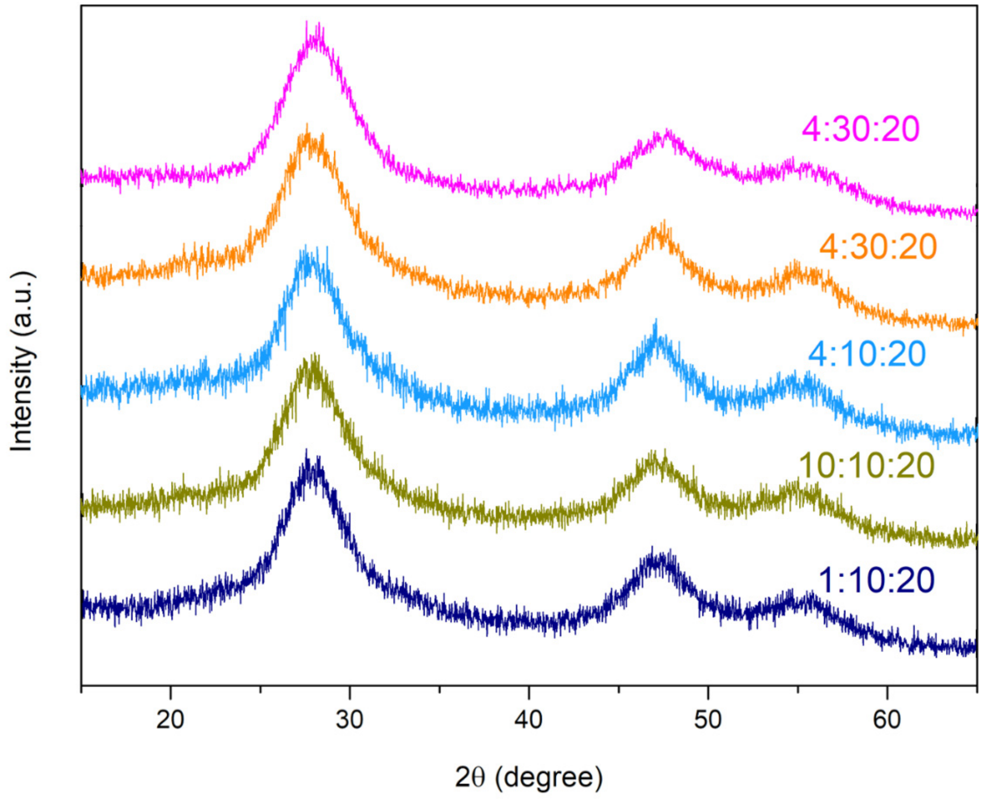

Figure S1. The XRD patterns of Cu-doped Zn-In-S QDs (The ratio in the graph represents the molar ratio of Cu:Zn:In).

3. Figure S2

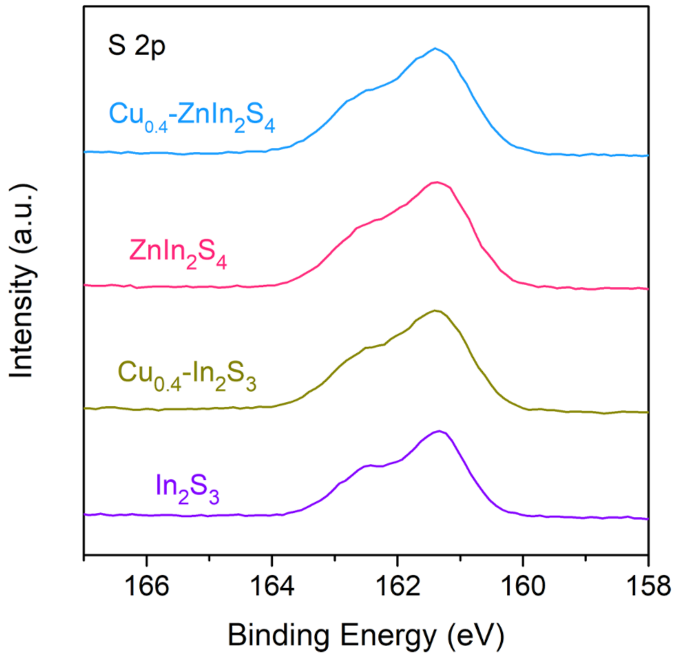

Figure S2. The high-resolution XPS spectra of S 2p of In_2S_3, $Cu_{0.4}$-In_2S_3, $ZnIn_2S_4$ and $Cu_{0.4}$-$ZnIn_2S_4$ QDs.

4. Figure S3

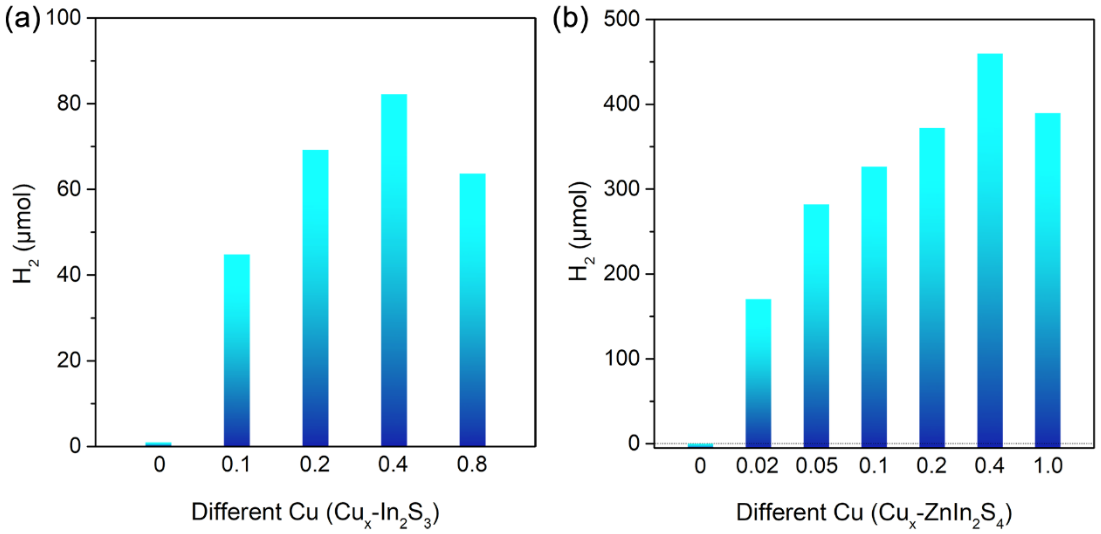

Figure S3. The photocatalytic H_2 evolution comparison of different contents of Cu doped (a) In_2S_3 and (b) $ZnIn_2S_4$ QDs.

5. Reference

1. Wang, F.; Wang, W.-G.; Wang, X.-J.; Wang, H.-Y.; Tung, C.-H.; Wu, L.-Z., A Highly Efficient Photocatalytic System for Hydrogen Production by a Robust Hydrogenase Mimic in an Aqueous Solution. *Angew. Chem. Int. Ed.* **2011,** *50*, 3193-3197.

2. Li, C.-B.; Li, Z.-J.; Yu, S.; Wang, G.-X.; Wang, F.; Meng, Q.-Y.; Chen, B.; Feng, K.; Tung, C.-H.; Wu, L.-Z., Interface-directed assembly of a simple precursor of [FeFe]-H_2ase mimics on CdSe QDs for photosynthetic hydrogen evolution in water. *Energy Environ. Sci.* **2013,** *6*, 2597-2602.

3. Han, Z.; Qiu, F.; Eisenberg, R.; Holland, P. L.; Krauss, T. D., Robust Photogeneration of H_2 in Water Using Semiconductor Nanocrystals and a Nickel Catalyst. *Science* **2012,** *338*, 1321-1324.

4. Li, Z.-J.; Li, X.-B.; Wang, J.-J.; Yu, S.; Li, C.-B.; Tung, C.-H.; Wu, L.-Z., A robust "artificial catalyst" in situ formed from CdTe QDs and inorganic cobalt salts for photocatalytic hydrogen evolution. *Energy Environ. Sci.* **2013,** *6*, 465-469.

5. Li, Z.-J.; Wang, J.-J.; Li, X.-B.; Fan, X.-B.; Meng, Q.-Y.; Feng, K.; Chen, B.; Tung, C.-H.; Wu, L.-Z., An exceptional artificial photocatalyst, Ni_h-CdSe/CdS core/shell hybrid, made in situ from CdSe quantum dots and nickel salts for efficient hydrogen evolution. *Adv. Mater.* **2013,** *25*, 6613-6618.

6. Chang, C. M.; Orchard, K. L.; Martindale, B. C. M.; Reisner, E., Ligand removal from CdS quantum dots for enhanced photocatalytic H_2 generation in pH neutral water. *J. Mater. Chem. A* **2016,** *4*, 2856-2862.

7. Huang, J.; Mulfort, K. L.; Du, P.; Chen, L. X., Photodriven charge separation dynamics in CdSe/ZnS core/shell quantum dot/cobaloxime hybrid for efficient hydrogen production. *J. Am. Chem. Soc.* **2012,** *134*, 16472-16475.

8. Wang, J.-J.; Li, Z.-J.; Li, X.-B.; Fan, X.-B.; Meng, Q.-Y.; Yu, S.; Li, C.-B.; Li, J.-X.; Tung, C.-H.; Wu, L.-Z., Photocatalytic hydrogen evolution from glycerol and water over nickel-hybrid cadmium sulfide quantum dots under visible-light irradiation. *ChemSusChem* **2014,** *7*, 1468-1475.

9. Gao, Y.-J.; Li, X.-B.; Wu, H.-L.; Meng, S.-L.; Fan, X.-B.; Huang, M.-Y.; Guo, Q.; Tung, C.-H.; Wu, L.-Z., Exceptional Catalytic Nature of Quantum Dots for Photocatalytic Hydrogen Evolution without External Cocatalysts. *Adv. Funct. Mater.* **2018,** *28*, 1801769.

10. Gao, Y.-J.; Yang, Y.; Li, X.-B.; Wu, H.-L.; Meng, S.-L.; Wang, Y.; Guo, Q.; Huang, M.-Y.; Tung, C.-H.; Wu, L.-Z., Self-assembled inorganic clusters of semiconducting quantum dots for effective solar hydrogen evolution. *Chem. Commun.* **2018,** *54*, 4858-4861.

11. Gao, Y.-J.; Li, X.-B.; Wang, X.-Z.; Zhao, N.-J.; Zhao, Y.; Wang, Y.; Xin, Z.-K.; Zhang, J.-P.; Zhang, T.; Tung, C.-H.; Wu, L.-Z., Site- and Spatial-Selective Integration of Non-noble Metal Ions into Quantum Dots for Robust Hydrogen Photogeneration. *Matter* **2020,** *3*, 571-585.
